# Whole-genome sequencing surveillance and machine learning for healthcare outbreak detection and investigation: A systematic review and summary

**DOI:** 10.1017/ash.2021.241

**Published:** 2022-06-13

**Authors:** Alexander J. Sundermann, Jieshi Chen, James K. Miller, Elise M. Martin, Graham M. Snyder, Daria Van Tyne, Jane W. Marsh, Artur Dubrawski, Lee H. Harrison

**Affiliations:** 1 Microbial Genomic Epidemiology Laboratory, Center for Genomic Epidemiology, University of Pittsburgh, Pittsburgh, Pennsylvania; 2 Division of Infectious Diseases, University of Pittsburgh School of Medicine, Pittsburgh, Pennsylvania; 3 Department of Epidemiology, Graduate School of Public Health, University of Pittsburgh, Pittsburgh, Pennsylvania; 4 Auton Lab, Carnegie Mellon University, Pittsburgh, Pennsylvania; 5 Department of Infection Prevention and Hospital Epidemiology, UPMC Presbyterian, Pittsburgh, Pennsylvania

## Abstract

**Background:**

Whole-genome sequencing (WGS) has traditionally been used in infection prevention to confirm or refute the presence of an outbreak after it has occurred. Due to decreasing costs of WGS, an increasing number of institutions have been utilizing WGS-based surveillance. Additionally, machine learning or statistical modeling to supplement infection prevention practice have also been used. We systematically reviewed the use of WGS surveillance and machine learning to detect and investigate outbreaks in healthcare settings.

**Methods:**

We performed a PubMed search using separate terms for WGS surveillance and/or machine-learning technologies for infection prevention through March 15, 2021.

**Results:**

Of 767 studies returned using the WGS search terms, 42 articles were included for review. Only 2 studies (4.8%) were performed in real time, and 39 (92.9%) studied only 1 pathogen. Nearly all studies (n = 41, 97.6%) found genetic relatedness between some isolates collected. Across all studies, 525 outbreaks were detected among 2,837 related isolates (average, 5.4 isolates per outbreak). Also, 35 studies (83.3%) only utilized geotemporal clustering to identify outbreak transmission routes. Of 21 studies identified using the machine-learning search terms, 4 were included for review. In each study, machine learning aided outbreak investigations by complementing methods to gather epidemiologic data and automating identification of transmission pathways.

**Conclusions:**

WGS surveillance is an emerging method that can enhance outbreak detection. Machine learning has the potential to identify novel routes of pathogen transmission. Broader incorporation of WGS surveillance into infection prevention practice has the potential to transform the detection and control of healthcare outbreaks.

Whole-genome sequencing (WGS) for infection prevention has traditionally been used in reaction to a suspected outbreak, usually at the end of an investigation to confirm or to refute the presence of an outbreak. In contrast, WGS surveillance of selected healthcare-associated pathogens regardless of whether an outbreak is suspected can be used to identify outbreaks that are not detected by traditional hospital epidemiologic methods. High costs and needed infrastructure for implementation have been historic barriers to widespread use of WGS surveillance. However, the cost of WGS has fallen, and the expansion of genomic surveillance due to coronavirus disease 2019 (COVID-19) may enable healthcare institutions to establish WGS surveillance programs for other pathogens. Additionally, our work and studies from Australia have identified cost benefits to implementing a WGS surveillance program with effective intervention.^1^

Although WGS surveillance is effective in identifying transmission, it does not provide information on the responsible transmission route, which is crucial for interrupting an outbreak. Traditional epidemiologic methods for identifying where transmission occurs have relied on geotemporal clustering within the hospital, which is inadequate for identifying more complex patterns of transmission.^2,3^ Automated analysis of electronic health records (EHRs) creates an opportunity to use machine learning or statistical modeling approaches for determining the outbreak transmission routes identified by WGS surveillance.^4–8^ These automated approaches would assist hospital infection prevention departments by providing systematic methods to investigate outbreaks and identify transmission routes.

In this systematic review, we provide a summary of prior studies utilizing WGS surveillance in healthcare settings for outbreak detection, as well as the use of machine learning and statistical modeling technologies to identify transmission routes. The purposes of this review were to summarize the current literature in this field, to identify barriers to widespread implementation, and to synthesize current knowledge on this topic to help guide decision making about implementation of WGS surveillance.

## Methods

**Fig. 1.**
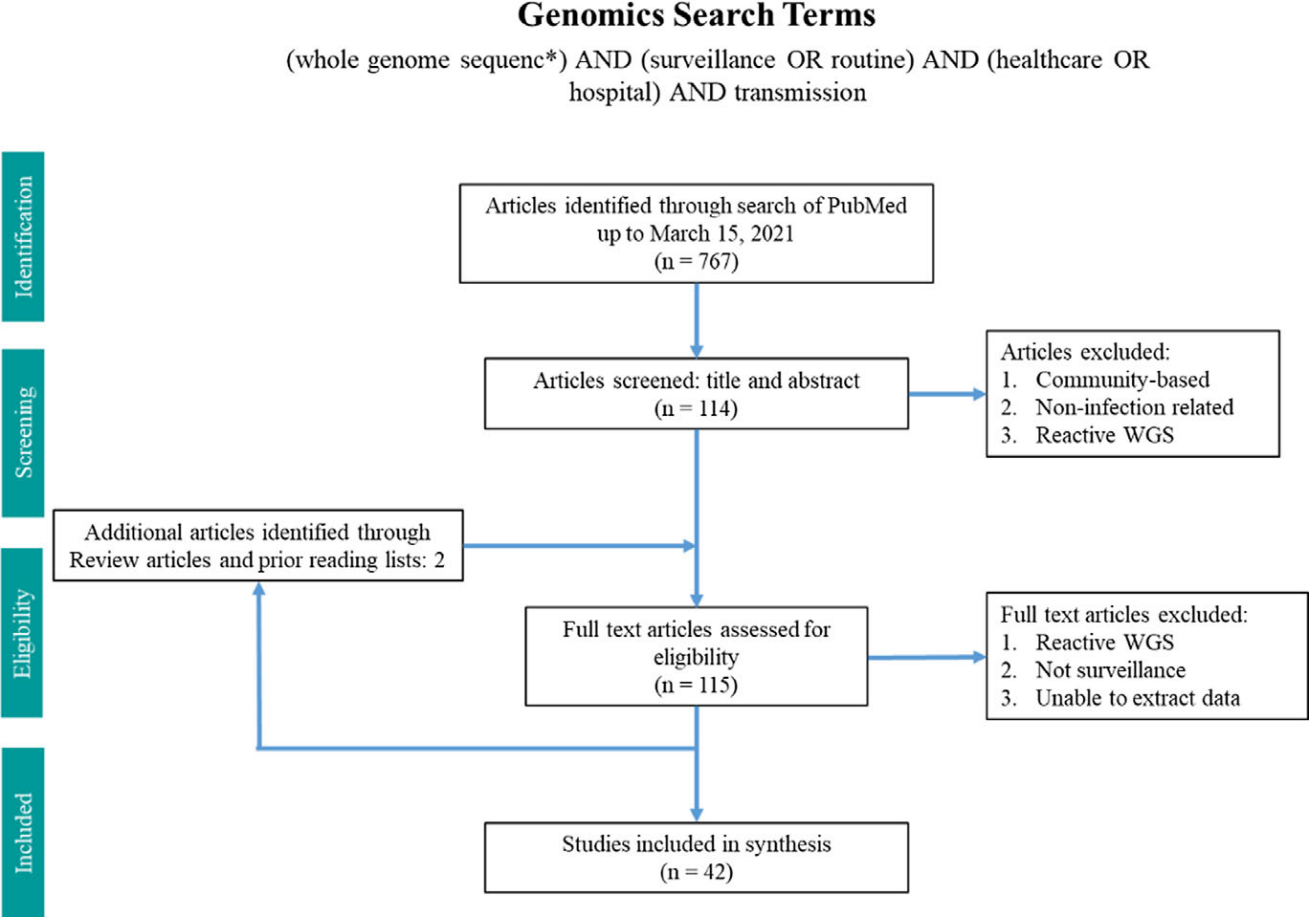
Search terms in PubMed for whole-genome sequencing surveillance.

**Fig. 2.**
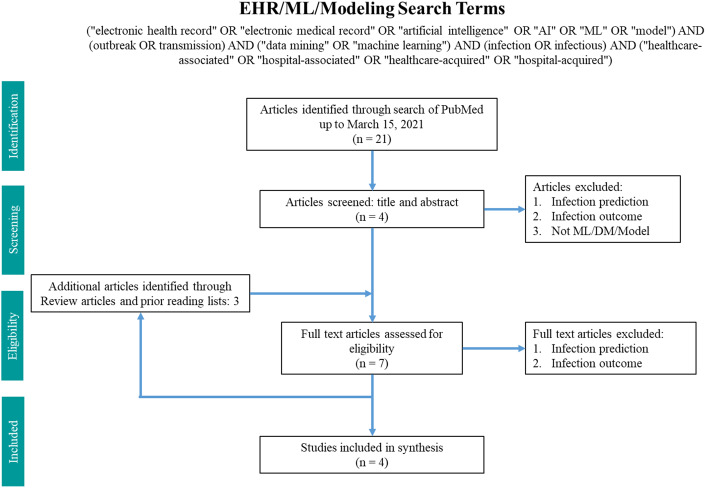
Search terms in PubMed for machine learning and modeling.

Two search terms were utilized in PubMed from inception until March 15, 2021 (Figs. 1 and 2). The WGS surveillance terms “(whole genome sequenc*) AND (surveillance OR routine) AND (healthcare OR hospital) AND transmission” returned 767 results. Article abstracts were screened to exclude studies that were solely community based, non–infection related, utilized non-WGS methods (eg, older molecular subtyping methods such as pulsed-field gel electrophoresis), or only utilized reactive WGS in response to suspected outbreaks. Genomic and epidemiologic data on organisms, number of isolates sequenced, percentage of isolates that were related, number of outbreaks, and epidemiological links were extracted and summarized. Articles were excluded if the data were not sufficiently detailed for extraction.

The machine-learning search terms utilized were “(“electronic health record” OR “electronic medical record” OR “artificial intelligence” OR “AI” OR “ML” OR “model”) AND (outbreak OR transmission) AND (“data mining” OR “machine learning”) AND (infection OR infectious) AND (“healthcare-associated” OR “hospital-associated” OR “healthcare-acquired” OR “hospital-acquired”)” and returned 21 results. Article abstracts were screened to exclude infection prediction and outcome studies. Data on the methodology and findings of outbreak and transmission detection models were extracted and summarized.

## Results

**Fig. 3.**
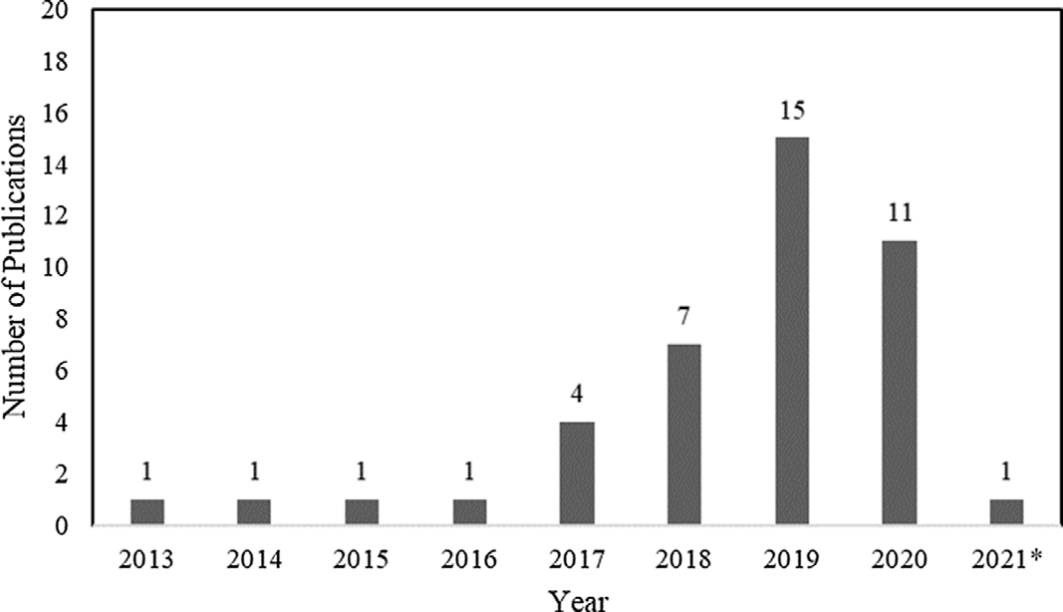
Summary by year of 42 whole-genome sequencing (WGS) surveillance studies in PubMed through March 15, 2021. *Through March 15, 2021.

In total, 42 articles on WGS surveillance were included in the final review.^3–5,9–47^ Among these studies, only 2 employed machine learning or statistical modeling to investigate transmission, which were also captured in the machine learning search. From 2013 to 2016, there was only one article per year, with a substantial increase thereafter (Fig. 3). Moreover, 12 studies were from the United States; 10 were from the United Kingdom; 5 were from Australia; 4 were from Germany; 2 were from Japan; and 1 study came per country came from China, Denmark, Finland, France, India, Italy, Netherlands, Spain, Sweden, and Thailand.

The duration of WGS surveillance varied substantially by study, with a median of 12 months and a range of 1–73 months (Supplementary Table S1). Only 2 (4.8%) studies were performed in real time; all other studies were performed retrospectively. Moreover, 39 studies included a single pathogen and 3 studies included multiple pathogens (Table 1). *Staphylococcus aureus* was the most commonly studied organism (12 studies, 28.6%), with 4 additional organisms present in >2 studies: 2 *Klebsiella pneumoniae*, 7 *Clostridioides difficile*, 6 *Enterococcus faecium*, and 3 *Pseudomonas aeruginosa*. Organisms selected for sequencing (eg, by anatomic site of infection, multilocus sequence type, and antibiotic resistance phenotype) were diverse across studies.

**Table 1.** Studies by Date, Organism, and Outbreaks Detected Utilizing WGS Surveillance

Year	First Author	Organism(s)	Type	Unique Isolates	Related, No. (%)	Outbreaks Detected	Epi Link, No. (%)
2021	Meredith^34^	SARS-CoV-2	…	299	159 (53.2)	35	35 (22)
2021	Miles-Jay^35^	*Escherichia coli*	ST131, H30	126	17 (13.5)	8	9 (52.9)
2021	Rose^39^	*Staphylococcus aureus*	Methicillin-resistant	56	15 (26.8)	7	7 (46.7)
2020	Berbel Caban^10^	*S. aureus*	Methicillin-resistant	224	33 (14.7)	8	…
2020	Cremers^12^	*S. aureus*	Methicillin-sensitive	84	40 (47.6)	14	0 (0)
2020	Gona^20^	*Klebsiella pneumoniae*	…	80	39 (48.8)	10	14 (35.9)
2020	Hammerum^23^	*K. pneumoniae*	…	103	36 (35)	13	11 (30.6)
2020	Marmor^31^	*Enterobacter cloacae*	…	63	7 (11.1)	1	0 (0)
		*Citrobacter freundii*	…		10 (15.9)	1	0 (0)
2020	Neumann^36^	*E. faecium*	Vancomycin-resistant	111	…	…	…
2020	Sundermann^5^	*P. aeruginosa*	ST27	882	31 (3.5)	10	1 (3.2)
2020	Sundermann^4^	*Enterococcus faecium*	Vancomycin-resistant, ST1471	439	10 (2.3)	…	1 (10)
2020	Tsujiwaki^44^	*S. aureus*	Methicillin-resistant	57	19 (33.3)	5	0 (0)
2019	Eigenbrod^14^	*Acinetobacter baumannii*	…	39	15 (38.5)	4	5 (33.3)
2019	Eyre^18^	*Clostridioides difficile*	…	299	43 (14.4)	6	20 (46.5)
2019	García-Fernández^19^	*C. difficile*	…	367	41 (11.2)	6	34 (82.9)
2019	Hall^22^	*S. aureus*	Methicillin-resistant	55	27 (49.1)	12	8 (29.6)
2019	Harada^24^	*K. pneumoniae*	Bloodstream infections	140	2 (1.4)	1	2 (100)
2019	Jakharia^26^	*C. difficile*	.	45	4 (8.9)	2	4 (100)
2019	Kossow^27^	*S. aureus*	Methicillin-resistant	.	8	1	0 (0)
2019	Mathur^33^	*K. pneumoniae*	Colistin-resistant	21	8 (38.1)	4	0 (0)
2019	Roy^40^	Influenza	A H1N1	36	5 (13.9)	2	2 (40)
2019	Sherry^41^	Enterobacteriaceae	Carbapenemase-producing	291	53 (18.2)	12	8 (15.1)
2019	Stenmark^42^	*S. capitis*	Bloodstream infections	46	12 (26.1)	6	12 (100)
2019	Sullivan^43^	*S. aureus*	Methicillin-resistant	141	28 (19.9)	4	2 (7.1)
2019	van Beek^45^	*K. pneumoniae*	Carbapenemase-producing, ST512	…	20	2	4 (20)
2019	Wang^46^	*Corynebacterium striatum*	…	91	18 (19.8)	6	3 (16.7)
2019	Ward^3^	*S. aureus*	…	953	85 (8.9)	28	65 (76.5)
		*E. faecium*	…	86	13 (15.1)	5	9 (69.2)
		*Psuedomonas aeruginosa*	…	118	2 (1.7)	1	2 (100)
		*K. pneumoniae*	…	100	0 (0)	0	0 (0)
2018	Auguet^9^	*S. aureus*	Methicillin-resistant	610	261 (42.8)	90	13 (5)
2018	Donskey^13^	*C. difficile*	…	66	12 (18.2)	4	4 (33.3)
2018	Houldcroft^25^	Adenovirus	…	43	6 (14)	2	0 (0)
2018	Kwong^28^	*K. pneumoniae*	Carbapenemase-producing	86	53 (61.6)	4	10 (18.9)
2018	Leong^29^	*E. faecium*	Vancomycin-resistant	80	10 (12.5)	2	3 (30)
2018	Martin^32^	*C. difficile*	…	640	227 (35.5)	.	69 (30.4)
2018	Wendel^47^	*A. baumannii*	…	36	20 (55.6)	2	2 (10)
2017	Coll^11^	*S. aureus*	Methicillin-resistant	1465	785 (53.6)	173	187 (23.8)
2017	Eyre^17^	*C. difficile*	…	652	128 (19.6)	…	…
2017	Gorrie^21^	*K. pneumoniae*	…	106	17 (16)	5	0 (0)
2017	Raven^37^	*E. faecium*	Bloodstream infections	293	93 (31.7)	6	.
2016	Elbadawi^15^	*K. pneumoniae*	Carbapenemase-producing	46	4 (8.7)	1	0 (0)
2015	Roach^38^	*Staphylococcus epidermidis*	…	178	56 (31.5)	10	…
		*P. aeruginosa*	…	44	7 (15.9)	3	…
		*E. faecium*	…	36	13 (36.1)	3	…
		*S. aureus*	…	118	4 (3.4)	2	…
		*E. faecalis*	…	72	6 (8.3)	3	…
		*Stenotrophomonas maltophilia*	…	58	2 (3.4)	1	…
2014	Long^30^	*S. aureus*	…	305	0 (0)	0	…
2013	Eyre^16^	*C. difficile*	…	957	333 (34.8)	.	152 (45.6)

**Fig. 4.**
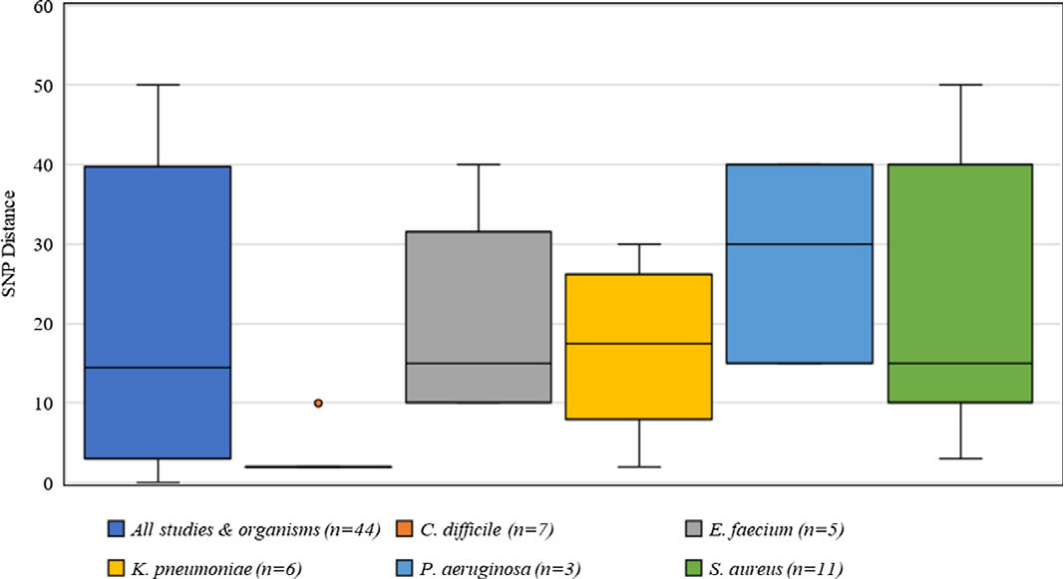
Distribution of single nucleotide polymorphisms (SNPs) for defining genetic relatedness from 42 studies.

Criteria for defining genetic relatedness were also highly variable between studies and were generally based on the number of single-nucleotide polymorphism (SNP) differences between genomes (Supplementary Table S1 online). Among organisms present in >2 studies, *C. difficile* had the most consistent SNP cutoff at 2 SNPs, and 1 study used 10 SNPs to identify related isolates (Fig. 4). Furthermore, *S. aureus* had the widest distribution of SNP cutoffs, ranging from 7 to 50 SNPs.

An analysis of the proportion of sequenced isolates that were determined to be genetically related to one another in each study revealed an average of 23.8% of isolates (range, 0%–61%). There were 525 outbreaks detected among 2,837 related isolates (average 5.4 isolates per outbreak). In addition, 41 studies (97.6%) found some level of genetic relatedness between the sequenced isolates.

We examined the methods employed to identify the responsible transmission routes for outbreaks that were detected by WGS. Overall, 35 studies (83.3%) restricted attempts to identify transmission routes to the same hospital unit during a defined period.^9,11–27,29–38,40–42,44–47^ Only 7 studies (16.7%) examined other possible routes such as medical procedures or healthcare workers.^3–5,10,28,39,43^

Several studies were notable for uncovering otherwise unidentified transmissions, which is the main goal of WGS surveillance. Sullivan et al^43^ were prompted by an outbreak in a neonatal intensive care unit (NICU) to retrospectively investigate all MRSA bloodstream infections for 16 months. Their investigation uncovered isolates related to the NICU outbreak from adult patients in a separate tower. Further investigation revealed shared ventilators between the adult unit and the NICU, which was believed to have caused transmission. Separately, Roy et al^40^ performed sequencing of influenza A H1N1for 6 months and found that traditional infection prevention practice falsely identified outbreaks, and WGS surveillance data were able to connect cases that were previously not believed to be epidemiologically related. Lastly, Berbel Caban et al^10^ utilized WGS surveillance of MRSA over 2 years and found multiple undetected outbreaks within 2 New York City hospitals. One cluster of 24 isolates from 16 patients spanned 21 months and 9 different hospital wards with patterns of shared healthcare workers. In this study, the authors emphasized the limitations of investigating only geotemporal clustering in outbreak detection and investigation.

Furthermore, 4 articles identified using the machine-learning search terms included in the final synthesis, 2 of which overlapped in the WGS surveillance search terms.^5,28,48,49^ Table 2 lists the methods and limitations of each study. One study utilized imputation of cultures to model transmission dynamics from environmental sink contamination, 2 studies used Bayesian methods to model transmission, and 1 study combined WHONET and SaTScan tools to detect outbreaks. In all of these studies, tools were implemented to supplement outbreak detection or investigation, yet the importance of manual or expert input to further investigate the transmissions or outbreaks detected was noted in each of these studies. In the study by Satchel et al,^49^ 45 outbreaks were identified, but only 6 were confirmed by an IP investigation. However, these researchers stated that the tool helped to streamline investigation efforts, which reduced time spent by the IP team.

**Table 2.** Studies Utilizing Machine Learning or Modeling to Detect outbreaks Or Transmission

Year	First Author	Machine Learning or Model Method	Utility and Findings	Limitations
2018	Lensing^48^	Imputation of clinical and environmental cultures to model transmission dynamics	Aided in targeted environmental cleaning to decolonize plumbing systems and reduce the risk of transmission of carbapenem-resistant Enterobacteriaceae based upon learned positivity	Requires expert knowledge of plumbing system and prior data on colonization
2018	Kwong^28^	Bayesian transmission modeling using Markov chain Monte Carlo	Assisted in transmission modeling of KPC-producing *K. pneumoniae* to determine if spread resulted from interfacility or intrafacility transmission	Does not provide specific details in sequence of transmission within a complex outbreak
2020	Sundermann^5^	Bayesian inference with case-control methodology to describe transmission sources	Scanned electronic health record and provided statistical output for possible transmission routes beyond but including geotemporal clustering	Requires robust mapping of electronic health record charge codes
2017	Stachel^49^	WHONET-SaTScan	Utilizes space-time permutation scan statistics to identify potential outbreaks	Low positive predictive value creating a high number of “false alarms”

## Discussion

In this systematic review, we synthesized studies that demonstrate the utility of WGS surveillance in finding cryptic outbreaks in healthcare settings. Nearly all studies (97.6%) identified outbreaks, but few (4.8%) utilized machine learning or statistical modeling methods to investigate transmission routes. WGS surveillance, while uncommon but increasingly utilized, aided infection prevention practice in these studies by uncovering outbreaks and enabling intervention.

Studies utilizing WGS surveillance have primarily relied on geotemporal linkage to identify transmission routes. Restricting investigations to geotemporal linkage fails to identify potential transmission by procedures that are performed in areas of the hospital other than patient nursing units or healthcare workers, as shown in some of the studies in this review. Some studies stated the limitations of relying solely on geotemporal parameters for identifying the transmission route for related isolates. Regardless, WGS surveillance enabled many of these studies to uncover substantial and significant previously undetected outbreaks that likely affected patient outcomes and associated healthcare costs.

Almost all of the studies reviewed were retrospective in nature, which limits the potential impact of WGS surveillance on healthcare epidemiology and infection prevention. If performed in real time, IP teams have an opportunity to perform an investigation (eg, audit practices, collect environmental cultures, and interview staff), which is not possible in retrospective studies. Furthermore, many studies focused on 1 pathogen, which is less sensitive for detecting outbreaks than WGS surveillance of multiple pathogens. For example, a single transmission route can lead to the spread of multiple pathogens.

Substantial investment and infrastructure are needed to establish real-time WGS surveillance. Healthcare institutions must have appropriate laboratory capacity, bioinformaticians, and genomic epidemiologists to interpret the data. A recent paper by Parcell et al^50^ discussed barriers to instituting a WGS surveillance program for outbreak detection from an economic and systemwide perspective. Indeed, it is often difficult to prove estimates of cost-effectiveness when considering prevention interventions, but 2 studies have demonstrated the cost-effectiveness of WGS surveillance programs.^1,7^

We identified very few studies on the utility of machine learning or statistical modeling methods for identification of outbreak transmission routes by WGS surveillance. In our experience, machine learning adds value in detecting transmission routes that do not involve geotemporal clustering such as invasive procedures, healthcare workers, outbreaks separated by unit, and outbreaks of longer duration.^5,6,8^ The use of machine learning in combination with WGS surveillance is clearly an understudied area of healthcare epidemiology and infection prevention. Barriers such as interoperability of electronic health records and adoption of WGS surveillance prevent the implementation of such programs. However, adoption of public health WGS surveillance for COVID-19 may expedite the use of this technology by healthcare institutions.

The combination of prospective WGS surveillance, EHR data, and machine learning has the potential to dramatically transform the paradigm of outbreak detection and investigation for infection prevention and control by identifying outbreaks quicker and enabling early intervention to halt transmission. This approach will both improve patient safety and reduce healthcare costs. However, healthcare institutional investment into establishing WGS surveillance programs will be key to expansion and implementation of this approach.

## Supplementary material

For supplementary material accompanying this paper visit https://doi.org/10.1017/ash.2021.241. click here to view supplementary material
